# Spatial-Orientation Priming Impedes Rather than Facilitates the Spontaneous Control of Hand-Retraction Speeds in Patients with Parkinson’s Disease

**DOI:** 10.1371/journal.pone.0066757

**Published:** 2013-07-03

**Authors:** Polina Yanovich, Robert W. Isenhower, Jacob Sage, Elizabeth B. Torres

**Affiliations:** 1 Rutgers University, Computer Science Department, Piscataway, New Jersey, United States of America; 2 Rutgers University, Psychology Department, Piscataway, New Jersey, United States of America; 3 Robert Wood Johnson Medical School, Piscataway, New Jersey, United States of America; 4 Indiana University, Medical School Indianapolis, Indianapolis, Indiana, United States of America; Hospital General Dr. Manuel Gea González, Mexico

## Abstract

**Background:**

Often in Parkinson’s disease (PD) motor-related problems overshadow latent non-motor deficits as it is difficult to dissociate one from the other with commonly used observational inventories. Here we ask if the variability patterns of hand speed and acceleration would be revealing of deficits in spatial-orientation related decisions as patients performed a familiar reach-to-grasp task. To this end we use spatial-orientation priming which normally facilitates motor-program selection and asked whether in PD spatial-orientation priming helps or hinders performance.

**Methods:**

To dissociate spatial-orientation- and motor-related deficits participants performed two versions of the task. The biomechanical version (DEFAULT) required the same postural- and hand-paths as the orientation-priming version (primed-UP). Any differences in the patients here could not be due to motor issues as the tasks were biomechanically identical. The other priming version (primed-DOWN) however required additional spatial and postural processing. We assessed in all three cases both the forward segment deliberately aimed towards the spatial-target and the retracting segment, spontaneously bringing the hand to rest without an instructed goal.

**Results and Conclusions:**

We found that forward and retracting segments belonged in two different statistical classes according to the fluctuations of speed and acceleration maxima. Further inspection revealed conservation of the forward (voluntary) control of speed but in PD a discontinuity of this control emerged during the uninstructed retractions which was absent in NC. Two PD groups self-emerged: one group in which priming always affected the retractions and the other in which only the more challenging primed-DOWN condition was affected. These PD-groups self-formed according to the speed variability patterns, which systematically changed along a gradient that depended on the priming, thus dissociating motor from spatial-orientation issues. Priming did not facilitate the motor task in PD but it did reveal a breakdown in the spatial-orientation decision that was independent of the motor-postural path.

## Introduction

Movements of the reaching family explicitly aimed at a target have been widely studied; see for example [1], [2], [3], [4], [5], [6], [7], [8], [9] among others. Biomechanical aspects of such motions are well understood, particularly for the forward trajectories deliberately aimed at attaining a goal. Often such motions also include a spontaneous hand retraction. This is an uninstructed withdrawal motion, not aimed at any specific goal and occurring largely beneath intentional awareness. The kinematics features of this portion of the reach-to-grasp action are largely underexplored. Yet the patterns of variability of this segment of the reach may offer some clues on the breakdown of the balance between voluntary and automatic control reported in Parkinson’s disease (PD) [10], [11]. We hypothesize here that the use of spatial-orientation priming [12] would impact the patterns of variability of the uninstructed retracting segments of the reach-to-grasp action differently from those of the forward segments. We further ask whether spatial-orientation priming would facilitate or impede the control of speed in patients with PD.

Under different spatial-orientation demands we explore the minute fluctuations (micro-movements) of the kinematics parameters from the movement trajectories. The micro-movements of the hand motions can be thought of as a form of re-afferent proprioceptive sensory feedback, contributing to the emergence of a motor percept that must be integrated with the visual percept of the object to be grasped. At the level of the continuous efferent motor output that is centrally driven, we can obtain a direct readout of the intrinsic changes that visual priming may induce in the micro-movements of action segments that are under explicit control. We may also detect those in the action segments that spontaneously occur without instructions. In particular we could assess such issues in movements with identical biomechanical goals as well as in movements with different biomechanics. The re-afferent information objectively measurable in the stochastic patterns of variability of the micro-movements may give us a handle on the potential contributions of peripheral proprioceptive input to the control of speed. This is currently largely under explored in PD.

We examine in this report the variations in the micro-movements not only as ***efferent*** motor output flowing from the central to the peripheral nervous systems. We also examine these variations as ***re-afferent*** proprioceptive input flowing from the periphery to the central regions as the participants make a motor decision that is driven by the spatial orientation of the object to be grasped. Here conscious and unconscious proprioceptive processes anatomically defined [13] would follow two very different routes through general somatic afferent (GSA) fibers targeting the cortical and sub-cortical/cerebellar regions respectively. We posit that typically intentional forward reaches may be under voluntary control using re-afferent proprioceptive feedback primarily along conscious proprioception routes, whereas the retractions may be more automatically guided by re-afferent proprioceptive feedback routed through sub-cortical/cerebellar pathways.

In PD such presumed dichotomy would break down. A possible scenario would be that in a given heterogeneous cohort of patients with PD such impairments would manifest differently in different patients. Some patients would manifest more voluntary control over the retractions than others. Such excess voluntary control –perhaps because the cerebellar proprioceptive feedback might be corrupted- would mask impairments in automatic control during the early stages of PD. Other patients would still maintain the typical dichotomy between voluntary-forward and automated-retracting motions that NC manifest in pointing behaviors [11]. The stochastic signatures of the motion variability from hand trajectory parameters would provide an objective measurement of such hypothesized patterns for typical and atypical cases along a gradient of performance. In particular, the statistical signatures of the withdrawing segments in patients with PD that were closer to those of NC would give us a sense of less degree of impairment in spontaneous, uninstructed motions, presumed here to contribute to central input via different afferent fibers than voluntary forward segments.

In one version of the task we let the participants choose the default hand orientation to match a given target orientation. In the other version we use orientation-priming [12]. Orientation-priming, a method commonly used in Psychology, prompts the participant with one specific way to orient the hand as if to grasp an oriented object. Because the upper limbs have many degrees of freedom (DoF) the object can be potentially grasped in more than one way while complying with the main orientation axis [7], [14]. The priming narrows down the set of affordances of an object to one specific orientation [15]. In this context the nervous system has unique strategies that have been well characterized geometrically and that typically remain conserved independent of changes in orientation, speed [7], [14] and postural demands [16] under abundant DoF. This is a problem originally posed by Bernstein who also noted that even when we master the unique motion trajectory, we do not perform it in the same way twice [17].

Orientation-priming has been found to typically facilitate the selection of a motor postural program under various conditions as well as to speed up decision-making processes [15], [18], [19]. Seminal work in the Psychological Sciences introduced biologically-plausible heuristics to solve postural and orientation-priming strategies [16], yet in PD such issues while explored in the perceptual domain [20], [21], have been much less explored in the motor domain, particularly in the context of motor variability conceived as a form of re-afferent proprioceptive feedback.

In PD at different stages of the disease both motor and non-motor impairments emerge [22], [23]. Patients with PD lose -in non-uniform ways- the delicate balance between different levels of voluntary control at different stages of the disease progression [10], [11]. These levels are also affected by the ways in which objects [24] and the context of a task [25] constrain movements of the reach-to-grasp family. Yet, motor and non-motor impairments are often confounded in PD. This new priming task may dissociate aspects related to higher motor complexity (such as the demands of additional physical rotations of the joints) from aspects related to higher cognitive-spatial demands involving decisions and selections of object-hand’s affordances.

We know that early on during the course of the disease, regions of the central nervous system (cortical and sub-cortical) are affected in PD but very little is known about the contributions of afferent input from the periphery. Hand movement variability, conceived here not only as efferent motor output but as re-afferent input as well, may help us elucidate possible different contributions of proprioceptive information to the breakdown of motor and non-motor aspects of this task. In the central anatomical regions, it is known that areas of the caudate and rostral putamen that mediate voluntary control [26], [27] are relatively spared from the degenerative process [28], [29], [30] so they could be in principle recruited to compensate for the loss of automatic control. In contrast the loss of dopamine in the posterior regions of the putamen–a region associated with automatic control [31], [32]–leads to deficits in automated arm-postural control when patients are off their dopaminergic medication, particularly between forward and retracting reaches [11]. Since the gradual dopamine depletion affects the automated motor performance and slows down the movements [33], [34], it is possible that increases in cognitive spatial-demands from continuously monitoring the movements might also contribute to bradykinesia. It is however unknown whether the breakdown would be gradual and also detectable in the stochastic patterns of kinematics parameters serving as re-afferent proprioceptive input from the periphery, or whether such statistical patterns would be unaffected by PD.

In this work we examine possible breakdown in proprioceptive input as a function of orientation-priming and ask if the stochastic patterns of velocity- and acceleration-dependent parameters during the retractions would be insensitive to the use of orientation-priming. Would orientation-priming facilitate or impede speed patterns in patients with PD as they decide between possible postures? Here we report a disruption in PD of the stochastic patterns of spontaneous withdrawing speed during uninstructed hand retractions. This impairment was only present during orientation priming and was independent of the motor-postural path. This dissociation may make orientation-priming a good candidate to assess in PD latent cognitive deficits before they explicitly surface.

## Methods

### Ethics Statement

All procedures were approved by the Rutgers University Institutional Review Board (IRB) in compliance with the Act of Helsinki. Patients signed a consent form voluntarily agreeing to participate in the study.

### Paradigm

We examined a heterogeneous cohort of 17 patients with PD that were at different stages of the disease with the main goal of blindly classifying them and then verifying if the blind classification was in any way consistent with the qualitative clinical reports. The patients were at the off-time of their medication (the time of the day when their medication had worn out). Patients were scheduled on an individual basis and accompanied by a care giver. None of the patients were treated for symptoms of depression. None had explicit cognitive deficits as assessed by their Neurologists. All patients were still active at work and held socially active lives at the times of their visit. Several patients were active in sports. One patient reported compulsive gambling in the past but the symptoms disappeared when switching to new medication. Three of the patients had undergone a Deep Brain Stimulation (DBS) procedure years ago and were tested with the DBS ON but off their medications as well. One of the DBS patients was still active teaching at a college. We were able to obtain records for all but 6. They were evaluated by the Neurologist using the Unified Parkinson’s Disease Rating Scale (UPDRS, average score was 26.18, ranging from 13 to 42). Patients for whom records are missing were diseased by the time that the tests were re-administered in our lab or had moved to a different state. We were no able to obtain previous records from other hospitals. However, the objective metrics that we use and report here are independent of subjective inferences made in observational inventories. The UPDRS scores provide a separate qualitative evaluation useful to gain an idea on the stage of the disease according to the gold standard of PD; but they in no way change our objective results. We also tested 9 normal controls (NCs), 5 males and 4 females ranging from 44–75 years of age.

**Figure 1:**
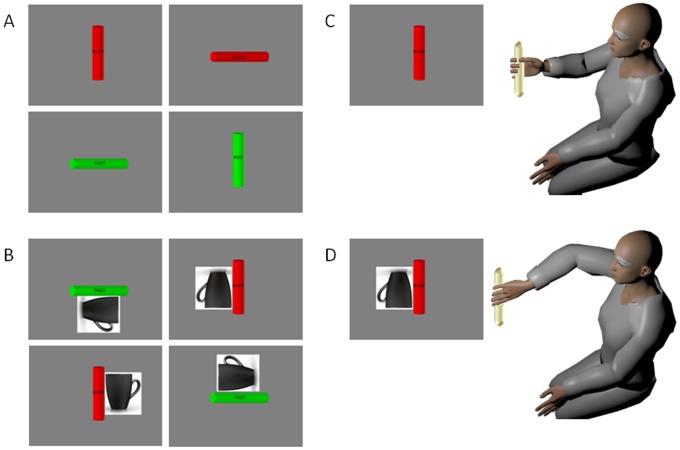
Priming Experiment to increase the cognitive load of a simple reach-to-grasp task. (A) Rods rendered in three dimensions were presented on the computer screen at 1 of 5 possible locations (shown here at the center location) at the four corners and at the center of the monitor. Color indicated the target speed (red-slow and green-fast). Speed was also labeled at the center of the cylinder. The target orientation could be horizontal or vertical. Because of redundancy in the degrees of freedom at multiple joints of the arm, each one of these oriented cylinders affords more than one arm-hand orientation. Subjects were free to choose the final orientation in the DEFAULT condition. (B) The primed condition instructed the subjects to use a particular target orientation while matching the hand-held cylinder to the simulated cylinder on the screen. The subjects were instructed to pick the orientation as though they were going grab the cup and drink from it. This instruction evoked a precise arm-hand orientation that was generally different from the DEFAULT one chosen by the subject in the first block. The primed-UP case required the same orientation as the DEFAULT but the primed-DOWN case required mental rotation to align the hand to the cup as if “picking it up to drink from it”. This orientation cue evoked rotations at the arm joints and at the hand that were unambiguously different from the DEFAULT and primed-UP cases. (C) An example of a DEFAULT arm-hand orientation evoked by the cylinder oriented vertically and positioned at the center of the screen. (D) The same canonical orientation of the cylinder evokes a very different arm posture and a different hand orientation during primed-DOWN.

Participants performed an orientation-matching task in a continuous forward-and-back loop while seated comfortably in front of a computer monitor. They held a rod in their hand resting it on the table. The task was to move the hand towards a virtual rod presented at 1 of 5 possible locations on a computer screen and orient the hand so as to match the principle axis of orientation of the virtual rod with the hand-held rod. Two canonical orientations were used: vertical and horizontal. On the rod the desired speed was instructed with text (**Fig. 1**), and also prompted with color (red for slow and green for fast).

In one case (DEFAULT) the movement was a biomechanical act devoid of the need to make a decision about possible hand configuration to match the target spatial orientation. The participants freely chose the final hand orientation. In the other version, primed-UP, orientation priming was used in such a way as to have identical biomechanics as those of the DEFAULT case. However the priming evoked decision making so the participant had to weight different possible hand configurations –all aligning with the spatial target orientation- but choose the one that the priming constrained. We used the picture of a coffee cup to instruct the desired target orientation and to prime the appropriate hand configuration. We then asked the participant “*gesture the final orientation of the hand as if reaching for and grasping the cup to drink from it*”.

**Figure 2:**
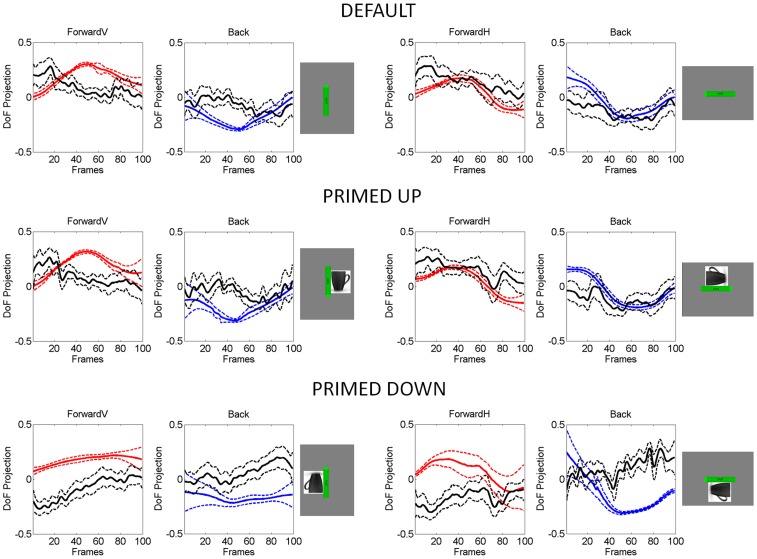
Similar biomechanical constraints for DEFAULT and primed-UP in vertical and horizontal cases contrast with different biomechanical demands between primed-UP and primed-DOWN. Traces are the averaged projection of the degrees of freedom of the arm along the dimensions relevant to the task goals and the dimensions incidental to the goals. These averages are taken across 100 trials for a typical representative and for 100 frames. Red continuous lines are task-relevant DoF forward. Blue are task-relevant DoF retractions and black are task-incidental DoF traces. Dashed lines are the standard deviation from the mean traces. During forward segments and retractions in the same loop the recruitment, release and balance of the degrees of freedom of the arm tend to markedly change as a function of task complexity.

A second version of the orientation priming was also used –opposite in orientation to the primed-UP (and DEFAULT) but still aligning the hand along the canonical principle axis of orientation. Because of the abundant DoF of the arm, it was possible to fully rotate the hand and match the cup handle in a completely different manner from primed-UP and DEFAULT while aligning it to the same canonical axis. This hand configuration required to end at a more complex orientation than the primed-UP (DEFAULT), one that required additional rotations and translations of the arm joint angles and of the hand (**Fig. 2**).

Five positions of the virtual rod were used, one in the center and four at each of the 4 corners of the monitor (**Fig. 1**). We used 20 trials in each block (2 speeds × 5 positions × 2 orientations) with 5 repetitions of each block. Endpoint accuracy was not enforced since we were interested in the patterns of variability of speed and acceleration *as the movement unfolded* in the continuous forward and back loop. Trials were randomized and balanced according to the combinations of position × orientation × speed within block.

**Figure 2** emphasizes that typically in this paradigm the interplay between the task-relevant and the task-incidental DoF changes as a function of task difficulty. The DEFAULT and primed-UP cases have on average similar postural rotational biomechanics. However, the primed-DOWN condition demands on average different joint angle recruitments. The joint angle decomposition of the postural excursions is from methods developed in [7] and [11]. These methods decompose 7 of the DoF of the arm into those which are relevant to the task goals and those which are incidental to it. Both the forward and retracting postural motions are shown for the vertical and horizontal cases of **Fig. 1**. In the forward motions the task-relevant DoF tend to dominate over the task-incidental components if the motion is more challenging –as in the vertical cases; then the hand retracts in “auto-pilot” mode, i.e. the task-incidental DoF tend to dominate. This is the typical behavior of the normal system for DEFAULT and primed-UP with identical biomechanics demands. By contrast the **Fig. 2** also shows the differences in the recruitment of the DoF for the primed-DOWN cases in relation to primed-UP and DEFAULT. There in both vertical and horizontal versions the task-relevant DoF tend to dominate over the task-incidental DoF.

Sample animations for all three cases are presented in the form of supplementary movies obtained from a representative patient and an age-gender matched control participant. The Motion Monitor (Innsport Inc.) software was used to render the motion captured kinematics and the Screen VidShot software was used to capture the movies.

### Apparatus

The movements were recorded by electromagnetic sensors (Polhemus Liberty, 240 Hz) mounted on Plexiglas and secured with Velcro at 11 locations. The output kinematics features of the movement trajectories from both hands were analyzed for comparison. Sensors were mounted on the forehead (1), trunk (2), both shoulders (2, acromial positions), both upper arms (2, brachial positions), both forearms (2, ante brachial positions), and both hands (2, on the top, manus position, opposite to the palms). The Motion Monitor (Innsport Inc.) was used to digitize the upper body and render it in real time for calibration purposes. Participants did not have visual feedback of their motions on the screen as they performed them. The stimuli shown to the participants in DEFAULT and primed conditions are shown in (**Fig. 1A**) and (**Fig. 1B**) respectively. The Motion Monitor recorded the positions and orientations of the various points of the limbs, trunk, and head. The PD patients are known to be affected asymmetrically. In this paper we focus on the trajectories of their most affected hand (which also happened to be the dominant one). Further analyses of the less affected non-dominant hand are reserved for another report.

## Metrics

### Motivation for New Statistical Metrics to Tackle the Heterogeneity of PD

**Figure 3:**
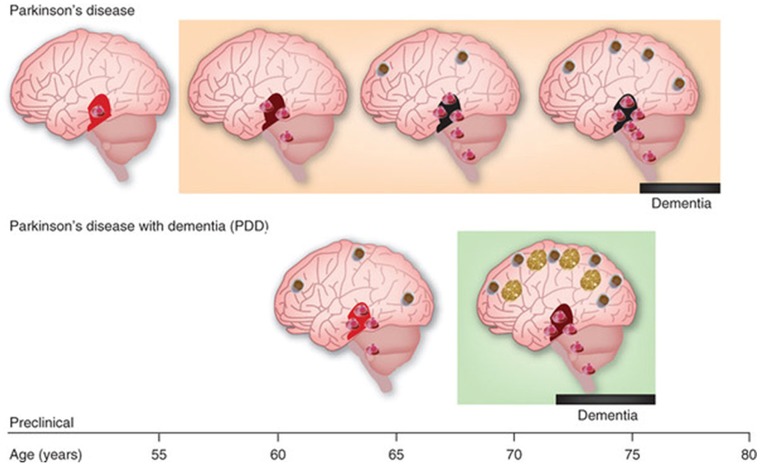
Examples of sub-types of Parkinson’s disease as a function of site of Lewy Bodies (LB) appearance in sub-cortical and cortical structures, density of LB and time course of spread. In all cases the presence of LB eventually leads to various stages of dementia and various subtypes of PD.

There is a general consensus -particularly among the clinical practitioners- that underlying the heterogeneity of Parkinsonism are different sub-types which current subjective observational inventories tend to obscure. In particular garden-variety Parkinson’s disease (PD) and Lewy Body Dementia (LBD) are considered the same disease in some circles as they both have the presence of Lewy bodies [35], [36], [37] albeit at different stages. In their initial stages three disorders are often confused: LBD, Multiple Systems Atrophy (MSA) and PD because they all manifest Parkinsonism. In the long run however, all three lead to dementia with severe non-motor (e.g. cognitive/memory) impairments (e.g. **Fig. 3**). It has been difficult yet highly desirable to sub-type disorders within the broad spectrum of PD early on to foretell differences and provide specific neuroprotective treatments. By now we know that how the disease progresses and how it affects the individual largely depends on ***where*** the disease begins [38], [39], [40], [41], [42], [43], [44], [45], [46], [47], [48]. Thus early formations of LB in the neocortex lead to dementia with marked cognitive/memory impairments [49]. When the LB form early in the substantia nigra and Basal Ganglia structures they destroy automatic control and eventually impede voluntary motions [50]. In the brainstem early formation of LB disrupts many vital autonomic functions that may rapidly lead to death (in MSA) [51].

In the initial stages the presence of LB generally leads to Parkinsonism (rigidity, bradikinesia or a marked reduction in movement amplitude, tremor in extremities at rest and postural instability), autonomic dysfunction (orthostatic hypotension, Gastro-intestinal problems, REM sleep disorders, etc.). It is imperative that what later becomes a highly differentiable diagnosis and therefore leads to a different treatment be identified earlier to prevent further damage and to slow down the progression and severity of dopamine depletion. At that early stage, when the differences are not as obvious to the human eye performing the inference-based diagnosis, micro-movement analyses may be a good predictive (putative) biomarker to blindly classify sub-types on the spectrum of many disorders –not just PD- and to provide personalized target therapies tailored to the individual’s behavioral patterns.

We propose here that one possible way of addressing the issue of heterogeneity is by first understanding the patterns of movement variability inherently present in the natural behaviors of people with PD on an ***individual*** basis.

Traditionally researchers in the social and medical sciences perform significance hypothesis testing under certain assumptions concerning the underlying probability distributions and assumed variance properties of a large sample. Often experimenters choose a priori large samples imposing as much homogeneity as possible in a particular type of sub-population (e.g. PD) so as to test a specific hypothesis (Significant Hypothesis Testing, SHT). The hypotheses are often chosen so as to unveil differences between and within the a-priori chosen groups with respect to some treatment, behavioral task, etc. In this context too much variability within or between samples are “the experimenter’s nightmare”, as it would wash out any experimental effects. Thus experimenters a priori homogenize the samples and design experiments that make people within a sub-sample look alike so as to attain strong statistical effects (supporting a highly sought after significant *p-value*). Here we instead exploit the variability inherently present in the motions of each person and take advantage of the rich statistical information that such variability offers. We look at the stochastic signatures of real time behavior, as the hand naturally moves towards and away from the target under difference experimental conditions.

Instead of handcrafting the sample to attain a certain *p-value*, we let the inherent variability of the sample reveal self-emerging patterns. Instead of assuming a common probability distribution for the sample, we actually estimate it empirically for each person, from the natural patterns of variability of each person. The behavior of each individual is examined across hundreds of repetitions so as to gain insights on the statistics of velocity-dependent parameters of the individual’s motions, as they continuously unfold. Across repetitions these stochastic signatures of the micro-movements contribute as re-afferent (movement- and position-sensed) proprioceptive input that may shift their statistical properties with context. They give rise to metrics of somatosensation that we can directly and continuously track at the efferent motor output to better understand the bi-directional flow between the central and the peripheral nervous systems. We propose that people with similar somatosensory-motor patterns may naturally cluster together. Likewise, people with dissimilar patterns may have different statistical signatures and be differentially situated. This approach exploits variability in a fundamentally different way than traditionally done and offers a dynamic picture of the individual’s natural behaviors useful to not only take a static snapshot of the individual during some experimental context, but to measure the person’s proprioception across different contexts as well.

In the next section on Distributional Analyses we explain the methodology that enables us to automatically label people according to their stochastic signatures of somatosensory-motor patterns when the motions are intended and when they are not.

### Distributional Analysis of Speed and Acceleration Maxima

The speed and acceleration profiles were obtained for each trial of the forward and retracting motions. We obtained the maximum value of the speed and acceleration in each segment. As in our previous work involving unconstrained arm motions [52] (**Figure S1**), the Gamma probability distribution family fit with 95% confidence the frequency distributions of maximum speed and acceleration values using maximum likelihood estimation (MLE). The histograms and estimation of bin size for the parameters of interest were obtained using in-house developed Matlab routines based on well-established algorithms for optimal estimation with 
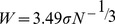
 [53] where *W* is the width of the bin, σ the standard deviation of the distribution (we used estimated standard deviation 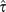) and *N* is the number of samples.

The Gamma probability distribution family was used to fit both each individual’s and the group’s speed and acceleration data. This is a two-parameter family of continuous probability distributions. Its probability density function is given by:
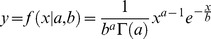
(1)with shape (a) and scale (b) parameters and the Γ function. By varying the shape and scale parameters, one can move from a Gaussian-like distribution to the Exponential distribution, with skewed distributions in between the two extremes (**Figure S1**). The random fluctuations from the movement acceleration of the forward and retracting motions of each participant were uniquely labeled by a point (a, b) in the Gamma-plane.

For each individual participant, we estimated the Gamma parameters and asked to what extent the scatter of points in the Gamma-plane segregated the forward and retracting segments of the continuous loop. To test if the stochastic signatures of acceleration maxima were different for each condition we fit a power relation to each scatter and compared the slopes and intercepts of each line. Similar slopes and intercepts would suggest that increases in spatial-cognitive load during spatial-orientation priming had no effect on the hand acceleration, so they would not impact the speed control. If on the other hand each condition had a systematic change of slope and intercept as a function of cognitive loads, this would support a link between effects on the patterns of speed variability and the changes of spatial-cognitive load above and beyond general slowdown (bradykinesia) known to characterize the movement speed of patients with PD. Motivated by similar questions we also performed a linear fit to the speed maxima data.

### Effects of Priming on the Movement Trajectories

The trajectories from the performing (dominant) hand were obtained for the DEFAULT and primed (UP and DOWN) conditions and were separated into forward and retraction segments. The primed-UP and DEFAULT cases were meant to evoke the same postural excursions and hand paths. The required movements for DEFAULT and primed-UP were biomechanically similar. Thus, any differences would have to be linked to the additional spatial-orientation priming constraint. Since fatigue could be also contributing to some effects, we examined the control of speed in retractions for the first five trials *vs*. the last five trials in the block.

In contrast to primed-UP, primed-DOWN was meant to evoke additional rotations at all joints. To evaluate whether the additional–and more complex–rotations changed the hand path curvature, a simple measure of linearity was used. The hand trajectory was resampled to obtain the spatial curve with a 100 points sampled at regular time intervals. The resampled trajectory was the same as the original but time-normalized. It was projected on the Euclidean straight line connecting the starting and the target positions. The normal distances from each point along the resampled hand trajectory to the corresponding point projected at a right angle on the straight line were obtained for each trial. We compared across trials the sum of the normal distances for DEFAULT and primed cases in each of the forward and retracting motion segments. Since the underlying distributions of this parameter were not Gaussian, non-parametric Kruskall-Wallis test was used to assess significance at the 0.01 alpha level.

### Statistics of the Speed Maxima and its Timing during Task-incidental Movements

We examined the statistics of the maximum speed values and of the time to reach those maximum speed values across patients with PD in relation to the NCs. Since the underlying distributions of these parameters were asymmetric, we used non-parametric Kruskall-Wallis ANOVA tests to determine if there were significant differences between patients and controls in the task-incidental retracting movements.

### Automatic Clustering of the Data

We used a traditional method of cluster analysis called *k-means* clustering [54]. This method aims to partition *n* observations into *k* clusters in which each observation is admitted in the cluster with minimum distance to its mean value. In our case *n* corresponds to the number of trials in each condition (100) and *k* corresponds to the two levels of instructed speed, fast and slow. Each point in the set of observations 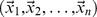 corresponds to a 2-dimensional real vector, on the plane 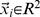. The components of each vector 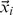 from trial *i* are the maximum value of the speed from the task relevant (forward) segment along the first dimension, and by the maximum value of the speed from the task incidental (backward) segment along the second dimension.

We ask if the *n* trials can be blindly partitioned into two sets corresponding to the slow and fast conditions 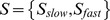 such that the within-cluster sum of squares is minimized, 
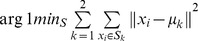
 where 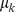 is the mean of points in the cluster 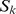.

Starting with a randomly chosen grouping into the two target groups for fast and slow, the algorithm uses an iterative refinement technique that alternates between two steps:

1. *Assignment step*, where each observation is assigned to the cluster with the closest mean, according to a distance metric. In this step we used the Euclidean distance metric. At each time step *t* of the algorithm, each point can exactly go into one of the clusters being formed as the points in the originally randomly chosen clusters of time step *1* are being shuffled around -until the assignments no longer change the clusters:

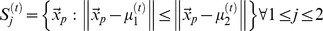
where *t* is the time step, 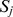 the number of clusters (fast and slow) being formed and
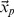
is the point being considered to ask in which cluster 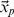
is the nearest neighbor to the mean of that cluster at that time step.

1. *Update step*, where the new means in the newly formed clusters are obtained and rendered as the new centroids of the observations that now form part of the new clusters:

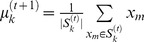
, where *k = 2* is the number of clusters in our case and *m* is the number of elements in the cluster formed at the previous step. The algorithm is said to converge when the clusters are stable in that their points no longer shuffle, i.e. when the assignments no longer change.

Once the algorithm automatically converges we compare the clusters thus obtained to the groups from the veridical data. The cluster assignment obtained from the *k-means* algorithm is compared for each data point with the veridical group assignment for this point. If the point comes from the estimated slow cluster but in reality it was a fast trial, it is counted as a mislabeled point. Otherwise it is counted as a correctly labeled point. This builds a metric of confusion for the case of misclassified points. Each patient has a number of misclassified points per condition, (DEFAULT, primed-UP, primed-DOWN) and this is rendered as a vector in three dimensions, where each axis corresponds to an experimental condition. We then plot the points in the three dimensional space and determine the plane of maximal separation of the misclassification across patients. This informs us about differences across patients between, e.g. DEFAULT and primed-UP conditions. We then mark using different colors any subgroups possibly emerging from the statistical *p-value* significance test above and see whether they overlap in the plane of maximal separation of the blind misclassification errors.

A possible scenario here is that misclassified clusters do not emerge at all, meaning that the subjects’ performance continuously maintained a good separation of the instructed speeds along forward and back motion segments. In this case the statistical results obtained through assessment of significance would be close to chance level and the separation thus obtained would be rendered dubious. Alternatively, if we found misclassified aggregates this would imply that some participants lost their control of speed in the retracting segments. This would imply different level of confusion between slow and fast trials between groups of subjects. We could then compare the subgrouping thus obtained with any previously emerging subgroups from *p-value* significance test. In such cases, if the subgroups from both sets of methods coincided, we would have consistently confirmed that the effects found –if any- were linked to cognitive deficits above and beyond motor deficits.

## Results

### Two Separable Classes of Motions: Forward Segments and Spontaneous Retractions

**Figure 4:**
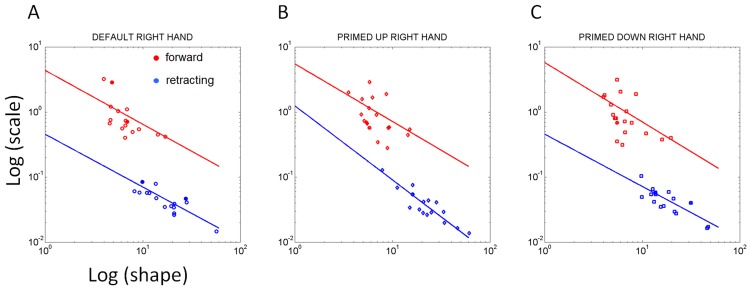
Individual stochastic signatures of variability for each patient separate the scatter in the Gamma plane representing the voluntary task-relevant (forward) and the spontaneous (retracting) task-incidental modes of acceleration control across all conditions. The cognitive load of the task systematically alters the slope of the retracting segments with spatial orientation priming.

The MLE of the (a, b) Gamma parameters for the values of the maximum acceleration in each individual case (taken across 100 trials per condition in each patient) were plotted on the Gamma plane. This is shown in **Fig. 4**: The red scatter corresponds to the task-relevant segments intentionally aimed at the target and under voluntary control. This scatter is well separated from the blue scatter, which corresponds to the spontaneous retractions incidental to the task. The two scatters emerging across subjects per condition discriminated well these two segments of the continuous reach-to-grasp task in both patients and controls. This confirmed that the linear hand-acceleration variability of the forward segment is in a different statistical class than the retraction, a prediction that the geometric decomposition of the joint angles had made (**Fig. 2**) based on the typical averaged variability of the joint angular rotations.

The log-log plots of these scatters for the empirically estimated shape-a and the scale-b parameters of the Gamma probability distribution were well fit by a power relation 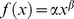 which yielded different exponents across conditions. This supported the notion of a systematic change in kinematics variability as a function of spatial orientation-priming condition, specifically during the retractions. Each point is a MLE of the (a, b) parameters of the Gamma distribution fit to the frequency histograms of the maximum acceleration across100 trials for each patient in each condition. We found different exponents for the DEFAULT and primed cases (as shown by the slopes) in the task-incidental retractions. Importantly the DEFAULT and primed-UP cases were designed with identical kinematics goals. These cases also had similar joint angular decomposition. Their biomechanics were similar (as shown by **Fig. 2**) across horizontal and vertical cases. The only difference here was the fact that the desired target spatial orientation was prompted in the primed-UP case but left unconstrained in the DEFAULT case.

DEFAULT condition fit for the voluntary trials was (0.48, −0.76) with 95%-confidence intervals [0.11, 0.86] and [−1.08, −0.45] for α and β respectively, R-square: 0.77 and RMSE: 0.01. In primed-UP the scatter was fit by (0.34, −0.62) with 95%-confidence intervals [−0.08, 0.77] and [−1.09, −0.15] for α and β respectively, R-square: 0.5 and RMSE: 0.01. In primed-DOWN the scatter was fit by (0.32, −0.56) with 95%-confidence intervals −0.028, 0.67] and [−1.00, −0.09] for α and β respectively, R-square: 0.45 and RMSE: 0.02.

The retraction trials yielded very different slopes and intercepts estimates as a function of cognitive load. In the DEFAULT case, the scatter from the retractions yielded (0.40, −0.75) with 95%-confidence intervals [0.03 0.83] and [−1.16, −0.35] for α and β respectively, R-square: 0.68 and RMSE: 0.013. The slope tilted in the primed-UP case, fit (0.20, −0.51) with 95%-confidence intervals [−0.06, 0.47] and [−1.00, −0.03] for α and β respectively, R-square: 0.39 and RMSE: 0.01. The primed-DOWN scatter was fit by (0.25, −0.5) with 95%-confidence intervals [−0.06, 0.47] and [−1.00, −0.03] for α and β respectively, R-square: 0.38 and RMSE: 0.01.

We emphasize that the slopes and intercepts of the aggregate scatters changed between the DEFAULT biomechanical condition and the priming cases. Even for the primed-UP case which was biomechanically similar to the DEFAULT case our methods unveiled changes in the random fluctuations of the acceleration maxima that traditional methods examining the averaged behavior would have missed. The comparisons within the orientation-priming variant (primed-UP *vs.* primed-DOWN) also showed systematic effects in the random fluctuations of the acceleration maxima as a function of the changes in spatial-orientation demands. Underlying these changes in acceleration during the retractions was a change in speed control that manifested differently for different patients in relation to the NC. We next objectively quantify such changes.

### Self-emergent Subgroups According to Speed Control of Retractions

All participants alike –NC and patients- maintained the instructed speed in the forward segment. However, unlike NC who maintained the speed of motion continuously in the forward and back loops, the patients with PD manifested a discontinuity in the speed control. This discontinuity manifested gradually along a gradient of severity as a function of the spatial-orientation priming complexity and as a function of the years since the diagnosis. The longer the patient had had the diagnosis, the higher the impairment in the speed control during retractions. Spatial-orientation priming did not facilitate the control of speed in PD. On the contrary it impeded the continuity and fluidity of this control in the spontaneous retractions of the reach-to-grasp motions.

For the primed-DOWN case the Mann–Whitney U test revealed two subgroups within the cohort according to the level of significance in statistical difference of the retracting speed maxima between fast and slow. The patients who showed no statistical differences at the alpha 0.05 level (denoted PD1) provided evidence for losing their distinction between fast and slow speeds in relation to patients who manifested statistically significant differences at the 0.05 level (denoted PD2). Recall here that the comparison includes hundreds of trials per patient.

The PD2 group was able to maintain the instructed speeds in the primed-UP condition during task-incidental retractions while the PD1 group lost their control over the instructed speed in this condition. Likewise, PD1 happened to have been diagnosed more than 6 years ago, while PD2 had been diagnosed on average less than 6 years ago. The diagnosis length was not a pre-designed hypothesis but rather a self-emergent pattern from the speed maxima separation manifested in the retracting motions during the more challenging primed-DOWN condition. **Table S1** boxed in the patients in PD1. All but 1 had over 6 years of diagnosis (average 8.28+/−2.21 years since diagnosis, range 3–20 years with average UPDRS 29.57+/−10.09, range 13–42). The PD2 group had below 6 years of diagnosis, average 2.7+/−1.7 years since diagnosis and average UPDRS 20.25+/−9.8, range 9–29). Including the DBS patients as part of the PD2 group or leaving them out did not change the reported differences between groups.

### Different Effects of Priming on the Movement Trajectories

Despite the similarities in required kinematics between DEFAULT and priming conditions, we found significant differences in the patients’ hand trajectories of these two conditions that were absent in the NC. The speed effects that gave rise to the two subgroups of patients above, also extended to the linearity metric detecting deviations from the straight line in the hand trajectories.

In the NC the priming conditions evoked significant differences in the bending of the wrist paths during both the task-relevant and the task-incidental segments of the loop (*Kruskal-Wallis* test, voluntary segment, median sum of deviations from straight line 4.43 cm, *p<10^−6^*, χ^2^ = 22.18, task-incidental segment, median 7.4 cm, *p<10^−12^*, χ^2^ = 50.15). These strong effects can be appreciated for a NC in the **Fig.2** for the primed-DOWN condition in which the hand underwent additional complex rotations to match the primed orientation. The insets of the figure also show the typical conservation of the instructed speed in both the forward and the retracting segments, albeit with higher variability in the task-incidental segments.

The differences in path curvature (trajectory bending) for primed-DOWN were also observed in some of the patients with PD, but those in the PD1 group had mixed effects of priming on the bending of their wrist trajectory. Their trajectories lost the distinction in curvature that NC had shown. In 12/17 patients the effects were comparable to those observed in the NCs. These patients showed similar ranges in the forward and retracting bending. The other 5 patients had modest systematic effects across DEFAULT and priming conditions, yet the bending of their trajectories was higher than NC (median forward 7.33 cm, and 13.7 cm in the retracting segments).

**Figure 5:**
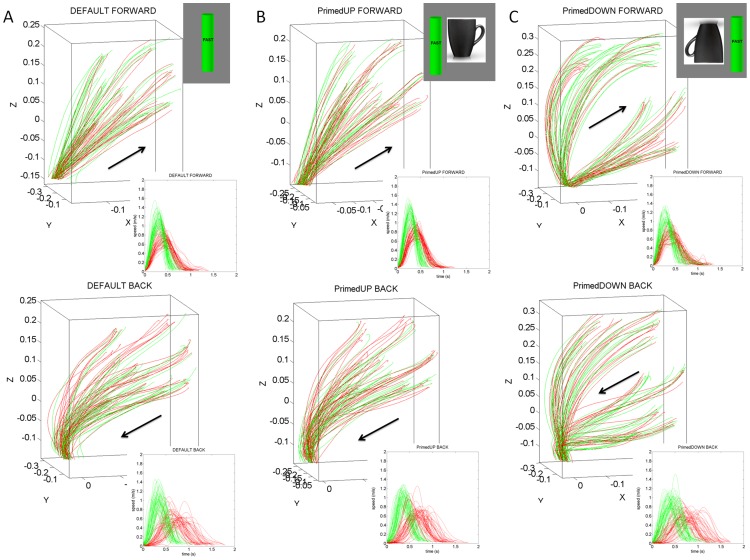
Effects of priming on the movement trajectories at the wrist in typical NC participant using two different levels of speeds randomly cued. (A) DEFAULT forward motion trajectories with corresponding speed profiles for slow (red) and fast (green) cases. Inset shows the actual stimuli on the screen priming the subject to match the orientation of the rod on the screen (vertical in this case) with the hand-held rod. Top are the forward paths and bottom are the retracting motions. (B) Primed-UP cases evoked similar final orientations as the DEFAULT condition. The inset shows the priming cup with handle next to the original rod. This figure is made from the right hand data and right hand stimuli for a right-handed person. (C) The primed-DOWN condition changed the trajectories in both the forward and retracting cases. The inset shows the priming condition where the arm and hand underwent complex rotations. The instruction was to match the orientation of the rod on the screen as if the hand were to gasp the handle of the cup to drink from it. Notice the dramatic differences in trajectories for all target positions. NCs maintain the instructed speed throughout the continuous forward-and-back loop.

**Figure 6:**
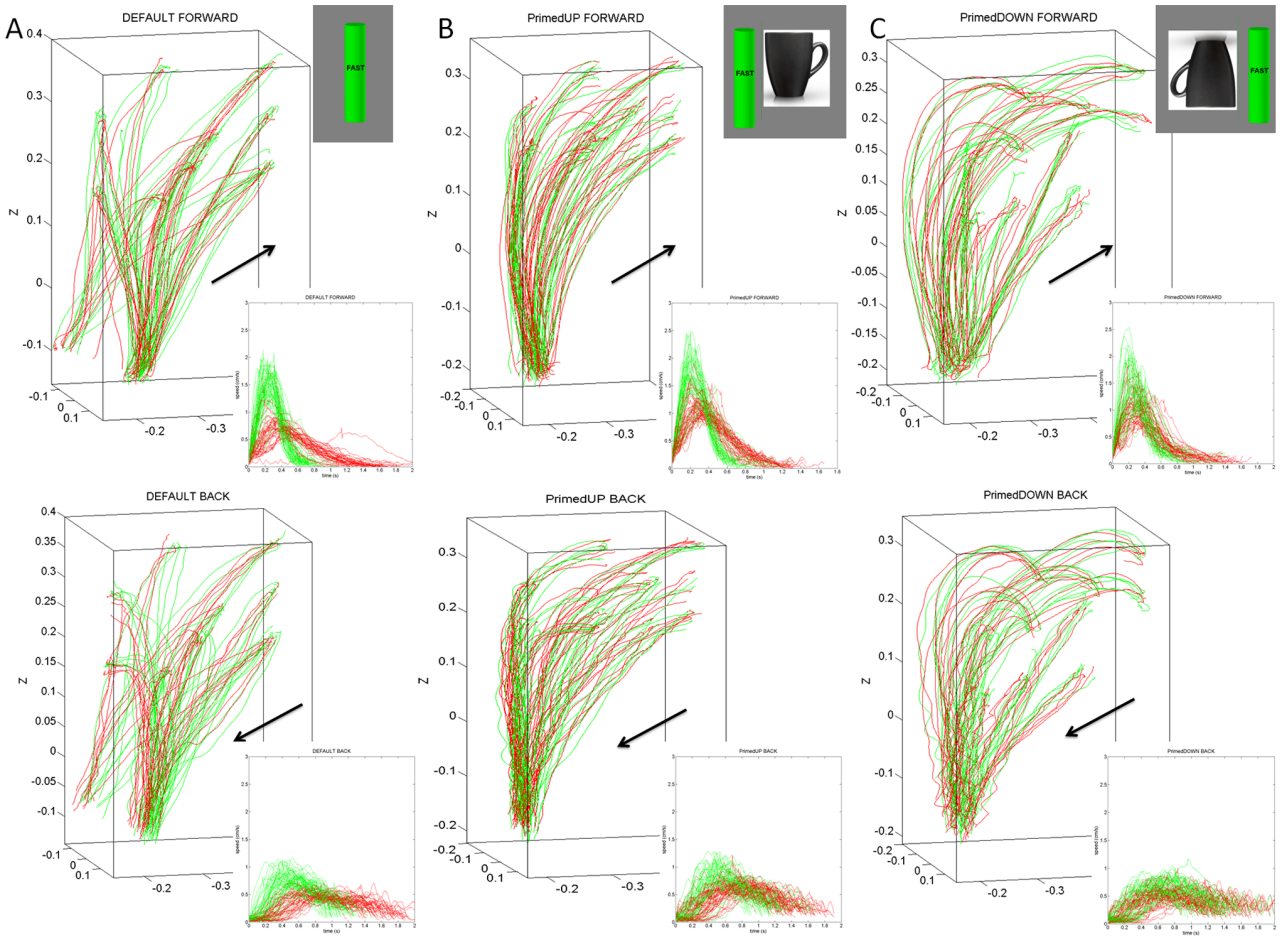
Effects of priming on the movement trajectories at the wrist in typical patient within PD2 group using two different levels of speeds randomly cued. (A) DEFAULT forward motion trajectories with corresponding speed profiles for slow (red) and fast (green) cases. Inset shows the actual stimuli on the screen priming the subject to match the orientation of the rod on the screen (vertical in this case) with the hand-held rod. Top are the forward paths and bottom are the retracting motions. (B) Primed-UP cases evoked similar final orientations as the DEFAULT condition. The inset shows the priming cup with handle next to the original rod. (C) Primed-DOWN condition changed the trajectories in both the forward and retracting cases. The inset shows the priming condition where the arm and hand underwent complex rotations. The instruction was to match the orientation of the rod on the screen as if the hand were to gasp the handle of the cup to drink from it. Notice the dramatic differences in trajectories for all target positions. Retracting speed profiles in the primed-DOWN condition in PD2 group do not have statistically significant differences for instructed fast and slow speeds.

**Figure 7:**
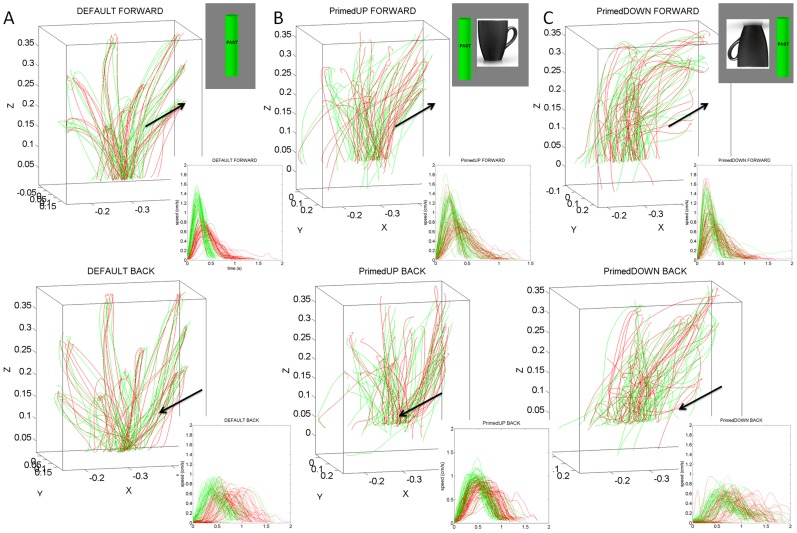
Effects of priming on the movement trajectories at the wrist in typical patient within PD1 group using two different levels of speeds randomly cued. (A) DEFAULT forward motion trajectories with corresponding speed profiles for slow (red) and fast (green) cases. Inset shows the actual stimuli on the screen priming the subject to match the orientation of the rod on the screen (vertical in this case) with the hand-held rod. Top are the forward paths and bottom are the retracting motions. (B) Primed-UP cases evoked similar final orientations as the DEFAULT condition. The inset shows the priming cup with handle next to the original rod. (C) Primed-DOWN condition changed the trajectories in both the forward and retracting cases. The inset shows the priming condition where the arm and hand underwent complex rotations. The instruction was to match the orientation of the rod on the screen as if the hand were to gasp the handle of the cup to drink from it. Notice the dramatic differences in trajectories for all target positions. Retracting speed profiles in both primed-DOWN and primed-UP condition in PD1 group do not have statistically significant differences for instructed fast and slow speeds.

Notice that across patients the differences in the geometry of the hand paths were not as revealing as those found in the temporal dynamics, which gave rise to the two subgroups within the cohort. The trajectories from a representative NC are depicted in **Fig. 5**. The trajectories from a representative patient in the better-off PD2 group are shown in **Fig. 6**. Notice the increased variability of the trajectories in general for the patients. In particular, notice the differences between the primed conditions and the increase in variability for the representative in the worse-off PD1 group shown in **Fig.7**.

Fatigue alone was not responsible for differences in the variability of trajectory kinematics with increases in cognitive load. The comparisons between the earlier and later trials within each condition showed that the effects of priming were as significant in the earlier trials as they were in the later trials. Earlier in the first five trials of the primed-UP block 15/17 patients were having difficulties maintaining the instructed speed during the task-incidental retractions even though the task requirements were similar to those for the DEFAULT condition where only 7/17 patients lost the task-incidental speed distinction. The mean *p* value across these patients in the earlier retractions of primed-UP was 0.36 (+/−0.3, range 0.016–0.99). Earlier trials were inseparable from later trials according to the instructed speed in the retractions during the primed-UP case. Later on in the last five trials of the block this lack of distinction was still present in the same patients. The mean *p* value of the later trials was 0.26 (+/−0.22, range 0.06–0.99).

By contrast during the DEFAULT case, where the required movement was biomechanically similar to the primed-UP movement the trend was different: only 7/17 patients lost the distinction between slow and fast during the 50 earlier trials. Comparison in each individual patient across each first 50 trials per condition gave (mean *p* value 0.36+/−0.23, range 0.09–0.63) and they kept these effects during the last 50 trials (mean *p* value 0.29+/−0.3, range 0.02–0.82). Within each of the DEFAULT and primed-UP cases, the inability to spontaneously control the retracting speed according to instruction remained despite the possible fatigue that repetitions could have induced.

Additionally the comparison between DEFAULT and primed-UP conditions did not yield significant differences between the 50 earlier and 50 later trials using early *vs.* late as the column-effects and the condition type as the row-effect in the non-parametric two-way ANOVA-Friedman’s test (Friedman’s test mean *p* values for the 17 patients 0.41+/−0.33, range 0.012–0.99, mean Chi-sq 1.78+/−2.05, range 0–6.3 ). Thus across patients we did not find evidence arguing for possible effects of fatigue.

### Distributional Analyses of Speed and Acceleration Maxima Based on the Patient’s Sub-grouping, Clustering and Blind Classification

**Figure 8:**
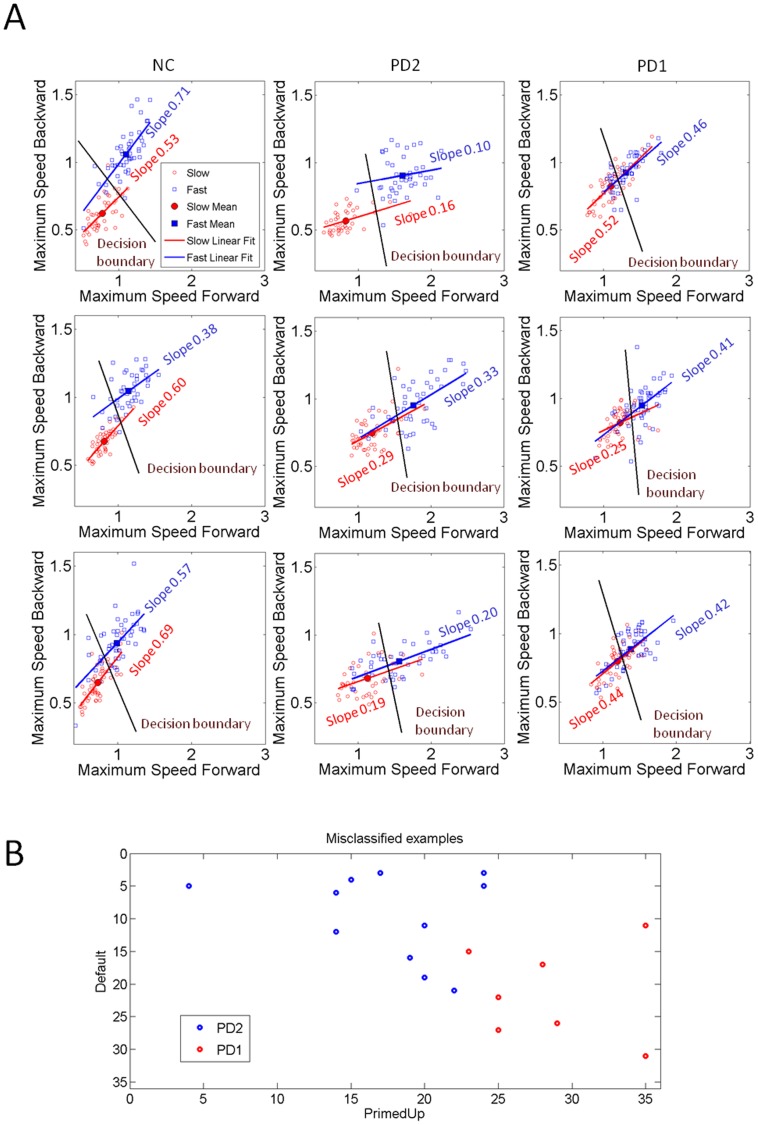
Self-emerging clusters and patient subtypes based on speed maxima (m/s). (A) Representative NC and patients from PD1 and PD2 subgroups grouped the speed maxima differently in the forward and retracting motions as a function of cognitive load condition. The NC maintained consistent separation across conditions in both portions of the pointing gesture yet patients consistently performed worse in the primed cases even though primed-UP was biomechanically equivalent to DEFAULT. PD1 performed the worst with no distinction in the primed cases. Slopes changed systematically with cognitive loads even for biomechanically similar DEFAULT and primed-UP motions. (B) Self emerging subtypes of misclassified trials from the blind clustering *k-means* separated exactly as the *p-value* statistics had predicted (see Methods and Results for details).

The clustering analyses revealed in the patient cohort systematic differences in the values of speed maxima as a function of cognitive load that confirmed the subgroups that had emerged from the statistical significance test. **Fig. 8** shows the scatters and the slope values of a linear regression fit. During the DEFAULT case NC and patients in the PD2 group clustered their slow and fast speed values within non-overlapping aggregates between the forward and the retracting cases. All NCs and patients maintained the instructed speed during the voluntary segments towards the target. Hence relatively large separations between the means of the fast and slow speeds along the horizontal dimension (forward segment) were quantified. In all NCs, the instructed speed was continuously maintained throughout the retracting motions as well. The differences between speed in NCs and patients were significant for both forward (median 1.42 m/s vs. 1.85 m/s, *Ranksum* test *p<0.11*×*10^−7^*) and retracting (median 0.99 m/s vs. 1.55 m/s, *Ranksum* test p<2.61×10*^−^* ^12^) motions.

In marked contrast to NC and patients in the better-off PD2 group, the patients in the worse-off PD1 group revealed overlapping of the scatters with no visible distinction between the slow and fast trials. Likewise during primed-UP patients in PD1 showed more mixture in their scatters that no longer distinguished fast from slow speeds, particularly along the vertical axis denoting the retracting segments. During primed-DOWN these deficits in speed control were exacerbated in the patients. Both PD1 and PD2 were affected by the more complex requirements of primed-DOWN (*Ranksum* test for each subject across 100 trials, mean *p* value 0.19+/−0.17, range 0.074–0.56).

The slope of the scatters systematically changed across conditions and between the two patient subgroups consistent with the statistical significance previously separating the two subgroups within the cohort (**Fig. 8**). This result ruled out a uniform slowdown of the motion as the exclusive link to the changes in speed. The conditions with different cognitive loads due to priming systematically modulated the relations between fast and slow speed differently for each patient type and for the NC’s.

There were significant differences between the value of the speed maxima of the slow and of the fast trials across all patients during the voluntary forward reaches (PD1 *vs.* PD2 median 0.55 m/s *vs.* 1.56 m/s (slow); 0.92 m/s *vs.* 1.79 m/s (fast)). Further details are in **Table S2**.

Both PD1 and PD2 groups maintained the instructed speed during voluntary forward motions in both primed and DEFAULT conditions (*Ranksum* test for each subject across 100 trials per condition, mean *p*<10*^−^* ^4^, +/−0.0016, range 10*^−^* ^15^+/−0.00054). Yet, both PD1 and PD2 groups could no longer maintain the instructed speeds during the task-incidental retractions for the most difficult primed-DOWN condition that required additional rotations of the arm joints The PD2 group maintained the instructed speed during the task-incidental retracting motions in both DEFAULT and primed-UP conditions. The maximum speed values were significantly different between the slow and fast speed trials (median 0.65 m/s vs. 1.15 m/s, χ^2^ = 215.05, *p<10^−48^*). The instructed speed in the PD1 group, however, was no longer significantly different when the target orientation was primed-UP (median 0.58 m/s vs. 0.61 m/s compared across 100 trials per patient χ^2^ = 0.86, *p>0.5*). The overall speed maximum was significantly different between the PD1 and PD2 groups for both the primed-UP and the primed-DOWN cases. PD2 were significantly faster (Kruskal-Wallis ANOVA χ^2^ = 216.24, *p<5.8*×*10^−49^*).

**Figure 9:**
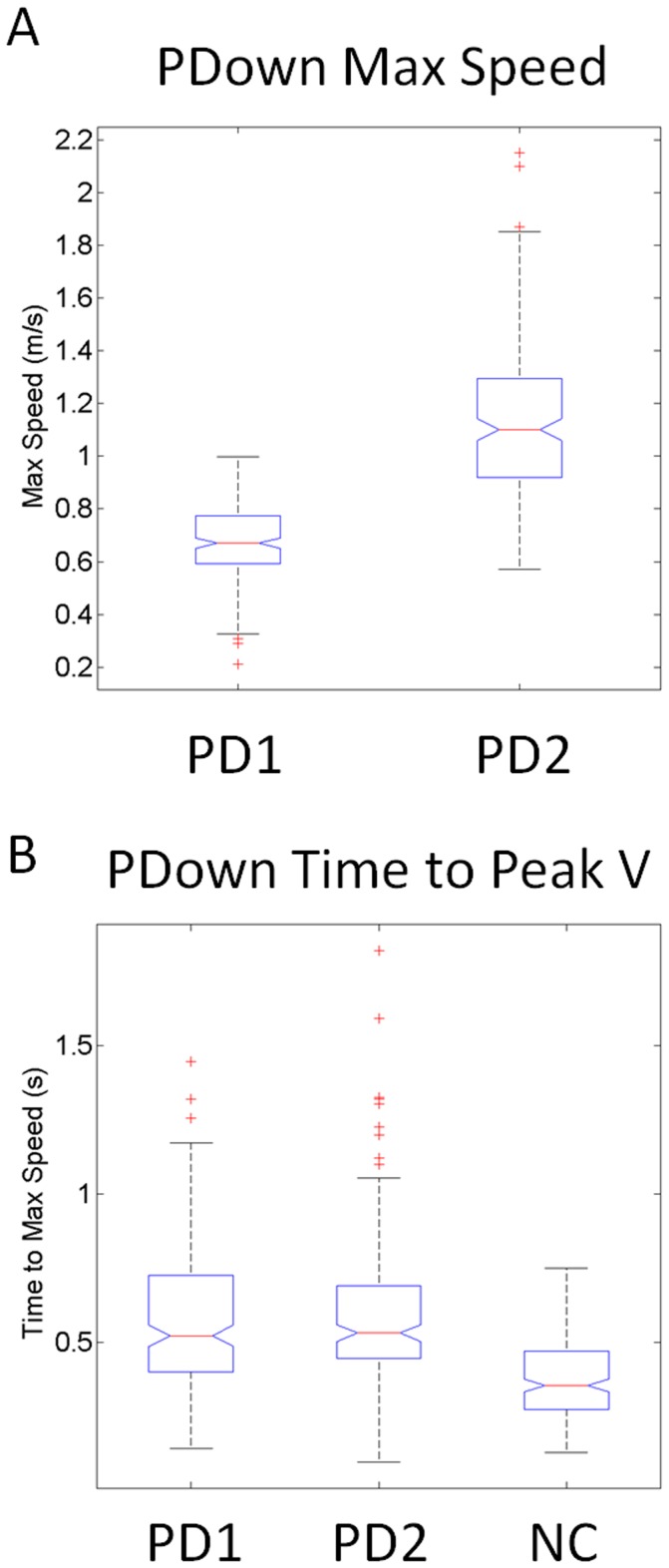
Effects of increasing task difficulty on the speed and timing of the reach by priming the final desired orientation such as to evoke complex mental rotation of the stimulus. (A) PD1 patients moved significantly slower than PD2 patients (median 0.65 m/s *vs.* 1.15 m/s, χ^2^ 215.05, p<10*^−^* ^48^) according to the values of the hand’s maximum speed returning from the target to the resting position. (B) The timing of the maximum speed was not significantly different in the two patient groups (median 0.52 s, χ^2^ 2.86, p>0.1) but both groups took longer to reach the velocity peak than NC’s did (median 0.35 s, χ^2^ 123.5, p<10*^−^* ^27^).

These differences in the distinctions of maximal backward speed values between PD1 and PD2 could not be accounted for by the time to reach the maximum speed during the task-incidental retractions, as these were indistinguishable between the two groups (median 0.52 s taken across 100 trials per patient, χ^2^ = 2.86, p>0.1) in marked contrast to the NCs (median 0.35 s also taken across 100 trial per participant, χ^2^ = 122.5, *p<10^−27^*). These marked differences in cognitive-load effects on speed variability between PD1 and PD2 are shown in **Fig. 9** for the maximal values of the speed (m/s) and for the time (s) to reach those values in the primed-DOWN cases.

### Different Stochastic Signatures of Variability of Speed Maxima

In this study we treat each patient of the cohort as a “case study” and densely sample his/her motion trajectories over hundreds of repetitions to estimate the underlying probability distribution describing the random parameters of interest. PD is a heterogeneous disorder because the progression of the disease is different even for people with the same number of years since their diagnosis and because the subjective observational inventories apply the same criteria to all patients. Thus any two patients with the similar UPDRS scores may differ tremendously even if they have had the diagnosis for the same number of years. The clustering methods used here permit the blind classification of different self-emerging subtypes within this cohort so as to better understand the underlying statistical properties of each PD self-emerging subtype. We extracted from the speed and acceleration variability across trials and conditions the stochastic signatures of each self-emerging subgroup according to the clustering analyses and also according to the blind misclassification analyses. This analysis confirmed that indeed these subgroups were in two different statistical classes according to the empirically estimated probability distributions of velocity dependent parameters.

Each of the patients in the subgroups contributes to the overall stochastic signature of the aggregate data making up that subgroup. The subgroups were not picked according to some feature. They rather automatically emerged from the data using blind *k-means* clustering. We also used confusion metrics to automatically reclassify the groups based on our veridical data and found 100% confirmation of the blind classification outcome and of the sub-grouping based on the *p-values* from the statistical comparison separating PD1 and PD2 patients according to the significance of the effects of cognitive load on speed compliance.

**Figure 10:**
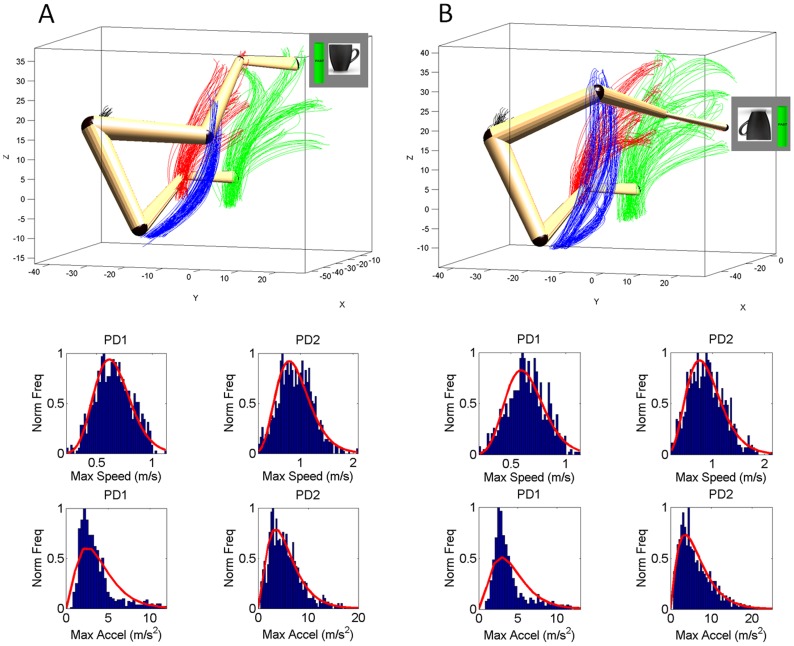
Normalized frequency distribution of the maximum speed and maximum acceleration values from the retracting primed motions in two groups of patients with PD. (A) Arm trajectories (shoulder, elbow, wrist, and hand) during the primed-UP condition similar to the DEFAULT case. The initial and final arm postures corresponding to the trajectories towards two randomly selected positions are superimposed. Patients in the PD1 group had a nearly symmetric distribution of speed maxima in the primed-UP condition where their retracting motions could no longer differentiate between the randomly instructed speeds. The group of PD2 was comprised of patients whose retracting motions could, on average, differentiate between instructed fast or slow speeds during the easier primed-UP cases. Their distribution of maximum speed values was skewed.

We found distinct stochastic signatures of variability in speed maxima between PD1 and PD2 (frequency histograms in **Fig. 10** **A–B**). Using MLE, the frequency distributions of the speed maxima from the aggregate of retracting trials from patients in each subgroup were well fit with 95% confidence by the parameters of the continuous Gamma probability distribution family. Patients in the PD2 group distributed in the ***skewed range*** of the Gamma distribution family (closer to NC) manifesting a multiplicative effect of the orientation priming on the overall variability of their retracting speed.

The MLE (a,b)-Gamma estimates for the PD2 was (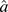 = 7.3, 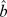 = 0.120) with [6.74, 8.02], [0.11, 0.13] 95%-confidence intervals. Bottom Panels of **Fig. 10** show skewed distributions of the maximum acceleration in the PD2 groups. For the maximum acceleration the MLE (a,b)-Gamma parameters were (2.75, 1.40), with confidence intervals [2.5, 3.02], [1.26, 1.56] for PD1 and (2.75, 1.98), [2.53, 3.0], [1.80, 2.17] for PD2.

In marked contrast to the PD2 and to the NC, the patients in the PD1 group were well fit during prime-UP by a ***symmetric*** ** ***distribution*** towards the normal range of the Gamma. This shows additive effects of cognitive loads on the variability of the retracting speed maxima. The MLE yielded shape (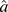 = 15.7) and scale (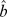 = 0.040) estimates for the maximum speed with [14.2, 17.3] and [0.03, 0.045] 95%-confidence intervals respectively.

The primed-DOWN condition evoked very different trajectories across all joints, an effect shown on the top panels of **Fig. 10B**. The arm was more abducted in primed-DOWN than in primed-UP (**Fig. 9A**) with longer and more variable elbow excursions that ended with a different orientation of the hand -as required by the priming stimulus. The MLE Gamma distribution parameters were (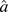 = 12.8), scale (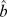 = 0.050) for the maximum speed with [11.61, 14.20] and [0.04, 0.055] 95%-confidence intervals respectively. The PD2 had very different MLE for the distribution of maximum speed (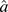 = 6.3, 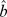 = 0.120) with [5.8, 6.91], [0.127, 1.153] 95%-confidence intervals.

Across patients in the PD1 group, the distribution of maximal speed values was nearly symmetric, and was centered at lower values than those of the distribution of the PD2 group, in congruence with the box plots shown in **Fig. 9A**. In PD2 the (a,b)-Gamma distribution parameters and confidence intervals were different from those of PD1. For PD1, the mle (a,b)-parameters had values in the Gaussian range of the Gamma. In contrast, the distribution in the PD2 case was skewed. The retracting motion of the patients in PD1 had excess predictability in relation to those of the NC and PD2 groups. It is possible that they were voluntarily monitoring their retracting motions which could also contribute to their being slower than those of the NC and PD2 patients.

**Figure 11:**
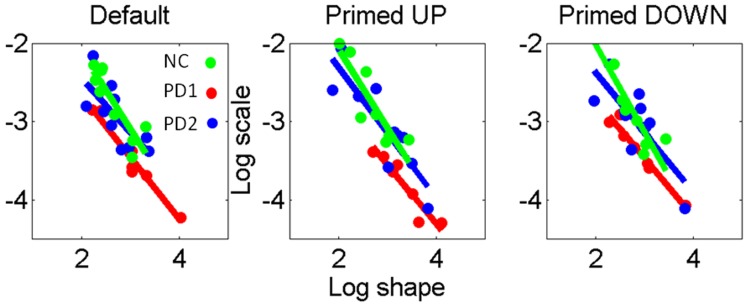
Individual stochastic signatures of variability for the retracting speed maxima in NC *vs.* patient types. Notice the changes in slope and intercept with changes in the cognitive load induced by the priming. Primed UP separates patients in PD1 maximally from the NC and from most of the patients in PD2. Primed DOWN shifts the stochastic signatures of the speed maxima for all participants, (details of the linear fit in Table S3).

The MLE values for the (a,b) parameters of the Gamma probability distribution family were determined for each subject. These are labeled in the Gamma planes of **Fig. 11** for the retracting motions according to subject type. Linear fit characterized differences in the scatter of points (participants) across priming conditions with different slopes and intercepts between the primed-UP and primed-DOWN cases. Table S3 lists the slopes and intercepts of the linear fit to the log shape-log scale of the Gamma plane. Note that in primed-UP the patients in the PD1 group (the worst performance) fall far from the NC and that most patients in the PD2 group fall closer to the NC than to the patients in PD1. Moreover the primed-Down condition shifts the stochastic signatures of all participants.

## Discussion

We studied a heterogeneous cohort of 17 patients at different stages of PD, treated with different medications, of different ages and of different sex. This is the typical profile that a clinician would see at his/her office any given day. Our main quest was whether in such heterogeneous sample we could blindly distinguish subtypes according to the stochastic signatures of the velocity- and acceleration dependent parameters of their hand motion trajectories. One of the main motivations for this question was that the fluctuations in stochastic signatures (micro-motions) that are inherently present in our continuous flow of movements can be conceived as a form of re-afferent (proprioceptive) input flowing from the peripheral to the central nervous system. In PD proprioceptive issues have been reported in homogeneous populations using traditional statistical techniques such as significant hypothesis testing. However, possible proprioceptive deficits tied to the sensing of movement variability had not been addressed [11], [55]. Here we were interested in the use of different methods from Statistical Physics and Machine Learning to tackle the heterogeneity of PD using a form of proprioceptive input, hand velocity- and acceleration- dependent variability. To this end we did not a priori homogenize the sample and searched for significant differences under a common (assumed) probability distribution for the cohort. Instead, for each participant we estimated from the empirical data, the probability distribution best describing the variability patterns (the micro-motions) of the kinematic parameters. Then we used clustering techniques to assess self-emerging subtypes within the heterogeneous cohort. In a last step we validated (whenever possible) the objective classification by taking into consideration the reported clinical scores from the subjective observational inventory (e.g. UPDRS). In the context of orientation-matching we examined possible shifts in the stochastic signatures of the hand velocity-dependent parameters with and without priming and used those statistical properties to classify PD severity.

We examined the continuous forward-and-retraction loop of the reach-to-grasp action. In this setting the retractions were not instructed. We were interested to find the extent to which the uninstructed motions were under voluntary control in PD. Given that across different tasks the spontaneous retractions typically have been more variable and noisier than the forward reaches under explicit voluntary control [11], [52], we hypothesized that if the uninstructed retraction segments were found to be more predictive, this would be indicative of more severity in PD, i.e. of more voluntary monitoring of otherwise spontaneous behaviors.

In PD the dichotomy between voluntary and automatic segments of the reach gradually breaks down so the retractions tend to also fall under voluntary control [11]. We reasoned that this presumed emergent deliberate control of the withdrawing motions in PD would be more evident as the disease progressed, rather than in its earlier stages. A subjective inventory like the UPDRS may or may not catch such subtle differences in movement variability but the objective stochastic metrics from the physical motions might.

We found support for our hypothesis: Forward movements intended to the target were found to be in a different statistical class than spontaneous hand retractions which were not aimed at any goal. The stochastic signatures of acceleration maxima differed between the forward and the retracting segments. Their frequency histograms were well fit by different underlying probability distributions of the Gamma probability distribution family. This finding held across NC and patients alike. However, patients manifested a gradual break down in the speed control of the retractions that manifested as a function of the type of spatial-orientation priming. In patients with PD the continuity in the speed of the forward and back loop was impaired as a function of spatial-orientation demands. In this regard we also answered the second question posed in this study regarding the use of orientation priming. We have found here that in PD the use of orientation priming hinders rather than facilitates motor performance. This impairment manifests along a gradient that worsens as the complexity of the spatial-orientation matching task increases. These effects blindly separate patients into subtypes that turn out to coincide with the number of years since their diagnosis.

We reasoned that retractions incidental to the main goals of a task may be differentially affected by concomitant goals of speed and spatial orientation as they fulfill a different (supportive) function than the goal-directed forward segment. Since motor and cognitive deficits are difficult to dissociate, here we specifically designed two variants of a task that had similar biomechanical demands with and without spatial orientation priming. Orientation priming typically facilitates the selection and execution of motor programs and speeds-up reaction time in psychological experiments [18], [19]. Here spatial orientation priming had the opposite effect. This effect was not due to motor impairments alone as they were also present under the similar biomechanical demands of the task in the DEFAULT and primed-UP versions. This result further confirmed and refined the separation that we had found between the goal-directed segments and the retractions according to the patterns of variability in acceleration maxima. The spatial orientation priming present in the primed-UP case dissociated in the patients aspects of the task which had similar postural demands from higher level spatial aspects requiring the decision of one compliant hand orientation over another. In the patients with PD we were able to further explore the breakdown in speed control during the priming condition.

The increase in complexity introduced by the prime-DOWN case further exacerbated the differences in speed control among the patients and gave rise to two self-emerging sub-types of PD severity within the cohort. Notice here that we did not hypothesize a priori the existence of these two different subgroups. They rather self-emerged from the stochastic patterns of variability in speed and acceleration maxima, a feature that traditional statistical methods that tend to homogenize the sample under study would have most likely missed. The patients in the better-off PD2 had a skewed distribution of speed maxima -closer to that of the NC’s- well fit by the Gamma probability distribution. The patients in the worse-off PD1, however, had a symmetric distribution underlying the random fluctuations of speed well fit by a Gaussian distribution with the MLE (a,b)-parameters in the normal range of the Gamma plane.

The unveiled differences in distributional properties underlying the speed and acceleration variability unambiguously separated the stochastic signatures of each self-emergent PD group, revealing fundamental statistical differences as a function of cognitive-spatial load. It is very important to note that, statistically speaking, the patterns of variability of their task-incidental speeds were not just significantly different. It is rather that their stochastic signatures across repetitions of the same motion shifted the parameter of the distributions into different statistical classes featuring skewed *vs*. symmetric probability distributions. These distributions have different multiplicative *vs.* additive statistical effects respectively [56], [57]. That is, the DEFAULT motion and its primed version (primed-UP) had different stochastic signatures despite having similar underlying kinematics requirements in the postural and in the hand domains. Moreover, differences between the underlying variability of the speed and acceleration of the primed-UP and primed-DOWN cases were also evident in the patients and manifested consistent gradual effects. This systematic modulation of the effects ruled out bradykinesia – a motor deficit characterized by an overall uniform slowness of their motions- as the sole source of the objectively quantified differences.

An emerging feature of the sub-types was that the effects that the increase in spatial-cognitive load had on the stochastic signatures of the hand velocity-dependent variability were more pronounced as the average time since diagnosis increased for the group. The emerging sub-types were confirmed by both the blind misclassification algorithm and the blind-cluster analyses that separated the speed data as a function of orientation-priming condition. Such effects would have been lost had we had homogenized the data.

During priming, the task-incidental retractions were significantly slower in PD1 than in the PD2 group. A potential confounding factor was fatigue. We ruled out fatigue however as the sole factor explaining the self-clustering results because earlier and later trials in the block both manifested these effects. Timing alone could not explain these differences either since both subgroups timed the peak velocity similarly. The differences in the random fluctuations of the peak velocity values during the retracting motions could be at least partly accounted for by higher curvature of the hand paths in the worse-off PD1 group than in the PD2 group. When curving more during the initial portion of the hand path, patients in the PD1 group increased the distance to be traveled to the peak velocity within the time period to reach the peak. This increase was systematic with the increase in spatial-cognitive load and therefore contributed to the systematic gradient effect in the lowering of speed of the retractions, a gradual effect also detected in the slopes of the scatters from the cluster analyses.

Orientation-priming normally facilitates human performance but here we captured strong deleterious effects on the uninstructed hand retractions of patients with PD. These effects emerged above and beyond motor requirements for the biomechanically similar versions of the task. We captured such effects using simple objective metrics of hand speed and acceleration variability. The new variant of the reach-to-grasp task was revealing of latent and systematic cognitive impairments along a gradient that worsened with the number of years since the diagnosis. We invite examination of the stochastic signatures of speed and acceleration during incidental, spontaneous segments of such habitual tasks to objectively predict forthcoming cognitive deficits, even during the earlier stages of the disease–when voluntary control can still successfully mask disruptions in the automated control of familiar acts.

## Supporting Information

Figure S1 **Schematic explanation using synthetic data of the individualized estimation of probability distributions and dynamic tracking of the stochastic signatures of kinematics parameters and their shifts using the continuous two-parameter Gamma family of probability distributions.** (A–C) The kinematic parameters of the hand motion trajectories are first obtained across hundreds of trials (e.g. for each segment we obtain the peak velocity, the peak acceleration, etc.) The frequency histograms of a given parameter are plotted (e.g. the peak velocity). We then use maximum likelihood estimation (MLE) to obtain the Gamma parameters (shape and scale) and fit the probability distribution. It has been our discovery that the Gamma family of probability distributions captures well all ranges of human statistical behavior [52], [58], [59], [60] ranging from (A) Exponential to (B) Skew to (C) Gaussian. (D) The three estimates are superimposed here as illustrative examples of possible scenarios. (E) The a-shape, b-scale parameters are plotted on the Gamma plane with 95% confidence intervals from the estimation process. Each point is representative of a measurement for one subject. (F) The shifts in the stochastic signatures can be dynamically tracked in real time to determine the individual’s rate of change in the stochastic signatures. They can also be longitudinally tracked to examine their progression as the system co-adapts exogenously- and endogenously-driven sensory patterns in different contexts. Shifts to the right towards the symmetric Normal range of the Gamma plane indicate more predictive patterns than shifts towards the Exponential range on the left of the Gamma plane (colors of the dots in E–F correspond to colors of the curves in D).
(TIF)

Table S1 **Demographic and clinical features of 17 PD patients tested in the “off” state (UPDRS unified Parkinson’s disease rating scale; motor subscale,** ***Am*** **Amantidine,** ***Don*** **donepezil,** ***Levo*** **Carbidopa/Levodopa,** ***Pra*** **pramipexole,** ***Ras*** **rasagiline,** ***Rop*** **ropinirole,** ***Sel*** **selegiline,** ***Tri*** **trihexyphenidyl).** Asterisk marks patients who underwent deep brain stimulation procedure. Boxed in are the patients in PD1 group who lost the task-incidental control of speed during both priming conditions. The non-boxed patients are in the PD2 group who only lost the spontaneous control of speed in the harder prime-DOWN condition. §Refers to number of years since diagnosis.
(DOCX)

Table S2 **Speed ranges (minima and maxima for each participant).**
(DOCX)

Table S3 **Linear fit to the log-log scatter of the Gamma plane in** **Figure 11** **.** Patient group PD1 separates maximally from the NC while most patients in PD2 are closer to the NC than to the patients in PD1.
(DOCX)

Movie S1 **Control_DEFAULT.avi: This movie contains the reach to grasp motion for one trial of the representative control for the default (easy) case.** The left panel shows the rendering of the digitization of the patient’s upper body from all sensors (including those on the left arm) but showing only the sensors as oriented axes relative to the world-axes. Sensors on the performing arm, head and trunk are shown on the actual locations along with the traces of the performing hand as the hand moves forward and back. On the upper right hand side panel we show the speed profile (m/s) for the continuous forward and back motions as a function of time (s). Motions normally took on the order of 500–800 ms for the fast variants and 1,000–1,500 ms for slow variants. Speed was below 2 m/s. The lower right hand side panel shows the corresponding acceleration profiles (m/s^2^). All of the supporting movies in format (.avi) were prepared with the open source software Screen VidShot and the Motion Monitor (Inn Sport Inc., Chicago, IL). The Motion Monitor was used to integrate motion captured with electromagnetic sensors (Polhemus Liberty, 240 Hz). Sensors are shown at a subset of the actual locations used to monitor upper body motions: the head, the trunk, the right scapula, the right upperarm, the right forearm and the right hand. The patient and control representative of the two main groups of participants were right handed.
(AVI)

Movie S2 Control_PRIMED_UP.avi: This movie contains a sample trajectory from a trial in the PRIMED-UP condition for the same representative control as the other Control_*.avi movies. This motion condition was biomechanically similar to the DEFAULT case but we used a coffee cup to prime the subject and constraint the various affordances of the cup’s handle to a specific one (coinciding with the DEFAULT in this case) for later comparison of the speed and acceleration variability. The format of the movie is the same as the description in Movie S1.
(AVI)

Movie S3 **Control_PRIMED_DOWN.avi: This movie contains a sample trajectory from a trial in the PRIMED_DOWN condition.** This motion condition was the most challenging as it primed a cup orientation to match that required additional rotations of the joints. This affected the trajectories of all sensors, including the hand. The corresponding speed and acceleration profiles changed and these changes were reflected over repetitions in the stochastic patterns of the speed and acceleration maxima of the continuous forward and back segments.
(AVI)

Movie S4 **Patient_DEFAULT.avi: This movie contains a sample trajectory from a trial of the DEFAULT condition from a representative patient.** This particular patient was a young (49 years old) fellow with a three-year diagnosis. This fellow is an athlete and suffers tremendously from the tremor at rest. His performance however turns remarkably smooth during the default motions. All panels are as in the other movies.
(AVI)

Movie S5 **Patient_PRIMED_UP.avi: This movie contains the performance from the representative patient during the PRIMED_UP condition.** This condition was similar to the default case in terms of biomechanical demands. However the constraints imposed by the priming visibly affected the motions. In particular his retracting motion speeds ceased to be smooth and the acceleration profiles also suffered.
(AVI)

Movie S6 **Patient_PRIMED_DOWN.avi: This movie contains the performance from the representative patient during the more challenging PRIMED_DOWN condition.** The format is the same as before.
(AVI)
