# Supplementary figures and images for: Spatial-Orientation Priming Impedes Rather than Facilitates the Spontaneous Control of Hand-Retraction Speeds in Patients with Parkinson’s Disease
Source: PLoS One. 2013 Jul 3;8(7):e66757. doi: 10.1371/journal.pone.0066757 (PMC3700979; doi:10.1371/journal.pone.0066757)

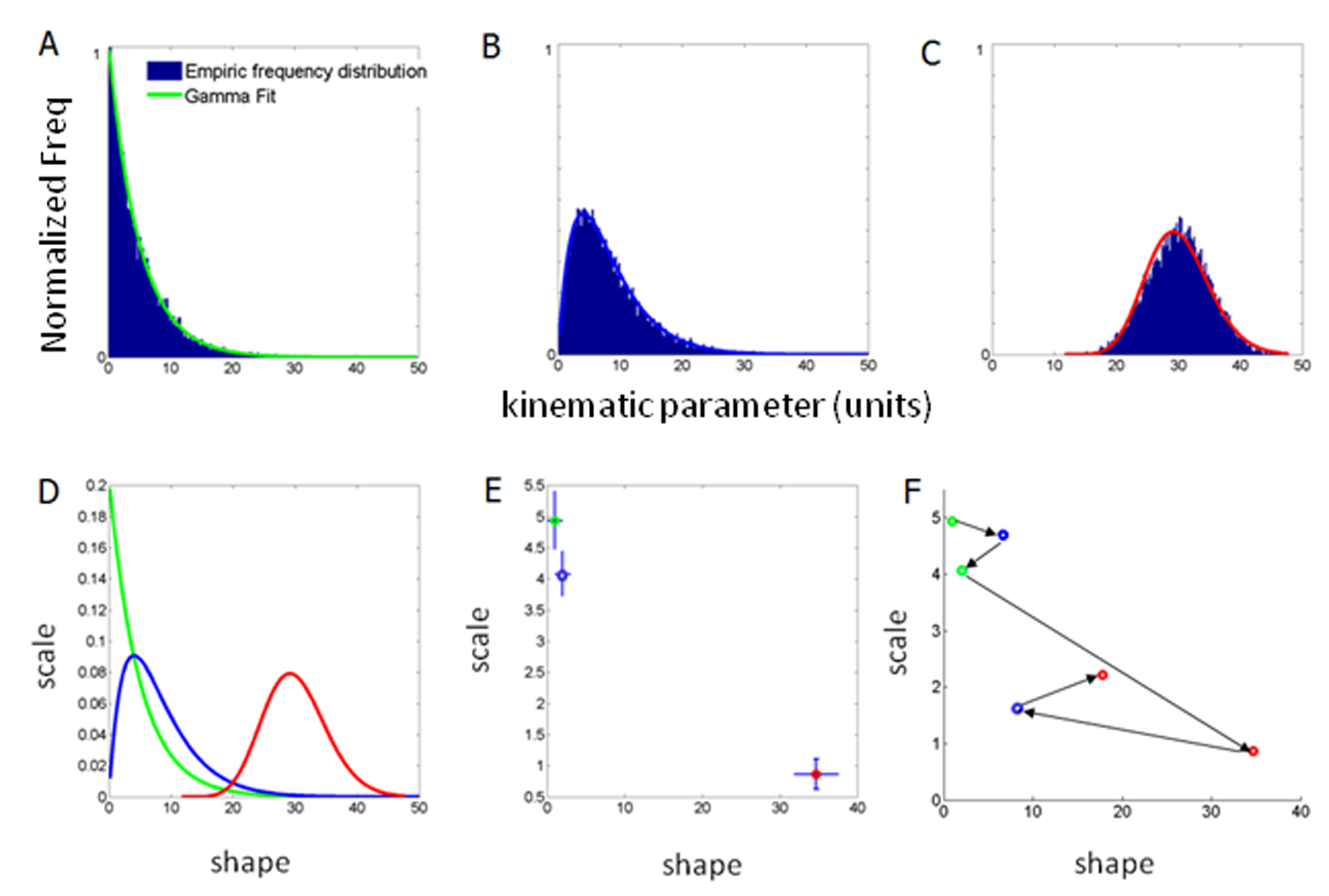

Supplement: Figure S1 — Schematic explanation using synthetic data of the individualized estimation of probability distributions and dynamic tracking of the stochastic signatures of kinematics parameters and their shifts using the continuous two-parameter Gamma family of probability distributions. (A–C) The kinematic parameters of the hand motion trajectories are first obtained across hundreds of trials (e.g. for each segment we obtain the peak velocity, the peak acceleration, etc.) The frequency histograms of a given parameter are plotted (e.g. the peak velocity). We then use maximum likelihood estimation (MLE) to obtain the Gamma parameters (shape and scale) and fit the probability distribution. It has been our discovery that the Gamma family of probability distributions captures well all ranges of human statistical behavior [52], [58], [59], [60] ranging from (A) Exponential to (B) Skew to (C) Gaussian. (D) The three estimates are superimposed here as illustrative examples of possible scenarios. (E) The a-shape, b-scale parameters are plotted on the Gamma plane with 95% confidence intervals from the estimation process. Each point is representative of a measurement for one subject. (F) The shifts in the stochastic signatures can be dynamically tracked in real time to determine the individual’s rate of change in the stochastic signatures. They can also be longitudinally tracked to examine their progression as the system co-adapts exogenously- and endogenously-driven sensory patterns in different contexts. Shifts to the right towards the symmetric Normal range of the Gamma plane indicate more predictive patterns than shifts towards the Exponential range on the left of the Gamma plane (colors of the dots in E–F correspond to colors of the curves in D). (TIF) [file pone.0066757.s001.tif]
