# Supplementary material for: Spatial-Orientation Priming Impedes Rather than Facilitates the Spontaneous Control of Hand-Retraction Speeds in Patients with Parkinson’s Disease
Source: PLoS One. 2013 Jul 3;8(7):e66757. doi: 10.1371/journal.pone.0066757 (PMC3700979; doi:10.1371/journal.pone.0066757)
Supplement: Table S1 — Demographic and clinical features of 17 PD patients tested in the “off” state (UPDRS unified Parkinson’s disease rating scale; motor subscale, Am Amantidine, Don donepezil, Levo Carbidopa/Levodopa, Pra pramipexole, Ras rasagiline, Rop ropinirole, Sel selegiline, Tri trihexyphenidyl). Asterisk marks patients who underwent deep brain stimulation procedure. Boxed in are the patients in PD1 group who lost the task-incidental control of speed during both priming conditions. The non-boxed patients are in the PD2 group who only lost the spontaneous control of speed in the harder prime-DOWN condition. §Refers to number of years since diagnosis. (DOCX) [file pone.0066757.s002.docx]

**Patient’s Demographics**

| **Subject** | **Gender** | **Age** | **UPDRS** | **Symptoms Duration (years)^§^** | **Meds** |
| --- | --- | --- | --- | --- | --- |
| **1** | F | 64 | 28 | 1 | *Levo* |
| **2** | M | 41 | 15 | 1 | *Levo; Pra* |
| **3** | M | 57 | 29 | 2 | -- |
| **4** | M | 49 | 42 | 3 | *Ras; Rop; Tri* |
| **5** | M | 70 | -- | 3 | *Levo* |
| **6** | M | 80 | -- | 3 | *Levo* |
| **7** | F | 67 | 9 | 3 | *Levo; Ras* |
| **8** | F | 77 | -- | 6 | *Levo; Pra* |
| **9** | F | 55 | -- | 6 | *Levo; Ras; Rop* |
| **10** | M | 77 | 31 | 7 | *Don; Levo* |
| **11** | M | 72 | 21 | 8 | *Levo; Pra; Ras* |
| **12** | M | 54 | -- | 9 | *Levo; Rop; Sel* |
| **13** | M | 60 | 35 | 10 | *Am* |
| **14** | F | 69 | 13 | 12 | *Levo; Pra; Ras* |
| **15*(DBS 47)** | M | 52 | 27 | 14 | *Am; Levo; Pra* |
| **16*(DBS 48)** | M | 59 | -- | 19 | *Levo* |
| **17*(DBS 70)** | F | 81 | 38 | 20 | *Levo; Pra* |

## Supplementary Table 1

Demographic and clinical features of 17 PD patients tested in the “off” state (UPDRS unified Parkinson’s disease rating scale; motor subscale, *Am* Amantidine*, Don* donepezil, *Levo* Carbidopa/Levodopa, *Pra* pramipexole, *Ras* rasagiline, *Rop* ropinirole, *Sel* selegiline, *Tri* trihexyphenidyl). Asterisk marks patients who underwent deep brain stimulation procedure. Boxed in are the patients in PD1 group who lost the task-incidental control of speed during both priming conditions. The non-boxed patients are in the PD2 group who only lost the spontaneous control of speed in the harder prime-DOWN condition. § Refers to number of years since diagnosis.
