# Supplementary material for: Spatial-Orientation Priming Impedes Rather than Facilitates the Spontaneous Control of Hand-Retraction Speeds in Patients with Parkinson’s Disease
Source: PLoS One. 2013 Jul 3;8(7):e66757. doi: 10.1371/journal.pone.0066757 (PMC3700979; doi:10.1371/journal.pone.0066757)
Supplement: Table S2 — Speed ranges (minima and maxima for each participant). (DOCX) [file pone.0066757.s003.docx]

## Supplementary Table 2 Speed ranges (minima and maxima)

|  |  | Default | | | |
| --- | --- | --- | --- | --- | --- |
|  |  | Forward | | Backward | |
| Patients |  | Min | Max | Min | Max |
|  | 1 | 0.522 | 2.140 | 0.461 | 0.996 |
|  | 2 | 0.236 | 1.587 | 0.415 | 0.961 |
|  | 3 | 0.196 | 1.209 | 0.172 | 0.919 |
|  | 4 | 0.503 | 2.016 | 0.460 | 1.919 |
|  | 5 | 0.616 | 1.785 | 0.651 | 1.506 |
|  | 6 | 0.785 | 1.641 | 0.742 | 1.706 |
|  | 7 | 0.506 | 1.245 | 0.509 | 1.252 |
|  | 8 | 0.375 | 1.055 | 0.337 | 0.870 |
|  | 9 | 0.777 | 1.666 | 0.598 | 0.994 |
|  | 10 | 0.440 | 1.654 | 0.321 | 0.898 |
|  | 11 | 0.220 | 1.159 | 0.183 | 0.832 |
|  | 12 | 0.603 | 2.100 | 0.487 | 1.882 |
|  | 13 | 0.256 | 1.232 | 0.275 | 1.091 |
|  | 14 | 0.408 | 1.464 | 0.293 | 0.987 |
|  | 15 | 0.411 | 1.534 | 0.312 | 0.998 |
|  | 16 | 0.352 | 1.163 | 0.323 | 1.199 |
|  | 17 | 0.290 | 0.949 | 0.299 | 0.901 |
|  |  |  |  |  |  |
| NC | 1 | 0.547 | 1.872 | 0.917 | 2.014 |
|  | 2 | 0.512 | 1.425 | 0.395 | 1.465 |
|  | 3 | 0.410 | 1.586 | 0.295 | 1.901 |
|  | 4 | 0.349 | 1.578 | 0.362 | 1.426 |
|  | 5 | 0.499 | 1.915 | 0.540 | 1.896 |
|  | 6 | 0.100 | 1.501 | 0.100 | 1.352 |
|  | 7 | 0.008 | 1.343 | 0.002 | 1.514 |
|  | 8 | 0.005 | 2.519 | 0.002 | 1.859 |
|  | 9 | 0.100 | 1.244 | 0.100 | 1.252 |
|  |  | primed-UP | | | |
|  |  | Forward | | Backward | |
|  |  | Min | Max | Min | Max |
| Patients | 1 | 0.765 | 2.508 | 0.580 | 1.286 |
|  | 2 | 0.442 | 1.827 | 0.345 | 0.984 |
|  | 3 | 0.315 | 0.968 | 0.227 | 0.849 |
|  | 4 | 0.685 | 2.130 | 0.655 | 2.100 |
|  | 5 | 0.589 | 2.096 | 0.522 | 1.500 |
|  | 6 | 0.785 | 1.641 | 0.742 | 1.706 |
|  | 7 | 0.399 | 1.374 | 0.515 | 1.325 |
|  | 8 | 0.272 | 1.117 | 0.363 | 0.999 |
|  | 9 | 0.884 | 1.926 | 0.535 | 0.990 |
|  | 10 | 0.237 | 1.370 | 0.380 | 0.708 |
|  | 11 | 0.270 | 1.161 | 0.199 | 0.837 |
|  | 12 | 0.419 | 1.812 | 0.466 | 1.927 |
|  | 13 | 0.265 | 1.362 | 0.294 | 1.033 |
|  | 14 | 0.752 | 2.082 | 0.615 | 1.562 |
|  | 15 | 0.458 | 1.586 | 0.363 | 1.424 |
|  | 16 | 0.365 | 1.258 | 0.377 | 1.128 |
|  | 17 | 0.303 | 0.949 | 0.344 | 0.844 |
|  |  |  |  |  |  |
| NCs | 1 | 0.977 | 2.611 | 0.866 | 1.998 |
|  | 2 | 0.512 | 1.434 | 0.509 | 1.290 |
|  | 3 | 0.369 | 1.540 | 0.509 | 2.990 |
|  | 4 | 0.417 | 1.413 | 0.428 | 1.208 |
|  | 5 | 0.509 | 2.039 | 0.667 | 1.919 |
|  | 6 | 0.100 | 1.349 | 0.100 | 1.170 |
|  | 7 | 0.013 | 2.462 | 0.004 | 1.159 |
|  | 8 | 0.011 | 2.049 | 0.002 | 2.196 |
|  | 9 | 0.100 | 1.374 | 0.100 | 1.325 |
|  |  | primed-DOWN | | | |
|  |  | Forward | | Backward | |
|  |  | Min | Max | Min | Max |
| Patients | 1 | 0.765 | 2.508 | 0.541 | 0.997 |
|  | 2 | 0.346 | 1.733 | 0.309 | 0.960 |
|  | 3 | 0.176 | 0.894 | 0.205 | 0.841 |
|  | 4 | 0.571 | 2.285 | 0.734 | 2.152 |
|  | 5 | 0.534 | 1.794 | 0.571 | 1.379 |
|  | 6 | 0.598 | 1.585 | 0.534 | 1.752 |
|  | 7 | 0.367 | 1.172 | 0.305 | 1.287 |
|  | 8 | 0.277 | 1.139 | 0.292 | 0.998 |
|  | 9 | 0.841 | 1.963 | 0.535 | 0.995 |
|  | 10 | 0.204 | 1.555 | 0.259 | 0.859 |
|  | 11 | 0.227 | 1.059 | 0.186 | 0.887 |
|  | 12 | 0.186 | 1.736 | 0.506 | 1.735 |
|  | 13 | 0.269 | 1.314 | 0.253 | 1.135 |
|  | 14 | 0.563 | 1.886 | 0.547 | 1.553 |
|  | 15 | 0.291 | 1.553 | 0.312 | 1.356 |
|  | 16 | 0.291 | 1.206 | 0.405 | 0.939 |
|  | 17 | 0.243 | 0.908 | 0.302 | 0.882 |
|  |  |  |  |  |  |
| NCs | 1 | 0.894 | 2.611 | 0.738 | 1.785 |
|  | 2 | 0.427 | 1.358 | 0.335 | 1.519 |
|  | 3 | 0.327 | 2.241 | 0.453 | 2.184 |
|  | 4 | 0.367 | 1.339 | 0.358 | 1.303 |
|  | 5 | 0.475 | 2.125 | 0.418 | 2.104 |
|  | 6 | 0.100 | 1.513 | 0.100 | 1.175 |
|  | 7 | 0.008 | 2.115 | 0.001 | 0.965 |
|  | 8 | 0.009 | 2.377 | 0.003 | 2.597 |
|  | 9 | 0.100 | 1.172 | 0.100 | 1.287 |
