# Supplementary material for: Spatial-Orientation Priming Impedes Rather than Facilitates the Spontaneous Control of Hand-Retraction Speeds in Patients with Parkinson’s Disease
Source: PLoS One. 2013 Jul 3;8(7):e66757. doi: 10.1371/journal.pone.0066757 (PMC3700979; doi:10.1371/journal.pone.0066757)
Supplement: Table S3 — Linear fit to the log-log scatter of the Gamma plane in Figure 11 . Patient group PD1 separates maximally from the NC while most patients in PD2 are closer to the NC than to the patients in PD1. (DOCX) [file pone.0066757.s004.docx]

|  | **Default** | **Primed UP** | **Primed DOWN** |
| --- | --- | --- | --- |
| **PD1** | R^2^ = 0. 9579,  RMSE = 0.0923,  (slope, intercept) = (-0.7925 -1.0692) | R^2^ = 0.8830,  RMSE = 0.1209,  (slope, intercept) = (-0.7565 -1.2752) | R^2^ = 0.9518,  RMSE = 0.0806,  (slope, intercept) = (-0.7457 -1.2183) |
| **PD2** | R^2^ = 0.5373  RMSE = 0.2585  (slope, intercept) = (-0.6972 -1.0414) | R^2^ = 0.4908,  RMSE = 0.4048  (slope, intercept) = (-0.8354 -0.6248) | R^2^ = 0.6107,  RMSE = 0.2892,  (slope, intercept) = (-0.7619 -0.8428) |
| **NC** | R^2^ = 0.7677  RMSE = 0.1992  (slope, intercept) = (-0.9901 -0.1178) | R^2^ = 0.7804,  RMSE = 0.2235,  (slope, intercept) = (-0.9996 -0.0711) | R^2^ = 0.8795,  RMSE = 0.2093,  (slope, intercept) = (-1.1156 0.2265) |

## Supplementary Table 3

Linear fit to the log-log scatter of the Gamma plane in Figure 11. Patient group PD1 separates maximally from the NC while most patients in PD2 are closer to the NC than to the patients in PD1.
